# Efficacy and safety of siponimod for multiple sclerosis: Protocol for a systematic review and meta-analysis

**DOI:** 10.1097/MD.0000000000015415

**Published:** 2019-08-23

**Authors:** Yumeng Song, Yongfeng Lao, Fuxiang Liang, Jing Li, Bibo Jia, Zixuan Wang, Xu Hui, Zhenxing Lu, Biao Zhou, Wei Luo, Bing Song

**Affiliations:** a Medical college of Soochow University, Soochow University, Suzhou; b School of Basic Medical Sciences, Lanzhou University; c Second Clinical Medical College of Lanzhou University; d Department of Cardiovascular Surgery, The First Hospital of Lanzhou University; e Public Health School of Lanzhou University; f First Clinical College of Lanzhou University, Lanzhou, China.

**Keywords:** meta-analysis, multiple sclerosis, protocol, siponimod, systematic review

## Abstract

**Background:**

Multiple sclerosis is the most common demyelinating disease of the central nervous system with serious social and economic burden. Siponimod is a sphingosine-1-phosphate receptor agonist, and clinical trials in the past decade have shown good prospects for the treatment of multiple sclerosis. But there is a lack of comprehensive understanding of the dose–effect relationship and safety in different subtypes of multiple sclerosis at present.

**Methods:**

We will perform a systematic review and meta-analysis of clinical randomized controlled trials to evaluate the efficacy and safety of siponimod in multiple sclerosis. We will search PubMed, EMBASE, Cochrane Library, Clinical Trials, Cochrane Central Register of Controlled Trials (CENTRAL) using a comprehensive strategy. The reference lists of the articles we select for inclusion will be checked to identify additional studies for potential inclusion. Two reviewers will review all literature independently. Upon inclusion of articles, another 2 reviewers will extract available data using a standardized form and assess the potential bias. Review Manager will be used to conduct data synthesis. There is no requirement of ethical approval and informed consent.

**Result:**

This is the first systematic assessment of siponimod for the treatment of multiple sclerosis. We predict it will provide high-quality synthesis of existing evidence for the efficacy and safety of siponimod for multiple sclerosis and a relatively comprehensive reference for clinical practice and clinical trials about siponimod to be conducted.

**Conclusion:**

The results of the systematic review and meta-analysis will provide updated evidence for the use of siponimod for multiple sclerosis.

**Registration:**

The systematic review and meta-analysis is registered in the PROSPERO international prospective register of systematic review (PROSPERO#CRD42018112721).

## Abstract 2

Supplemental Digital Content is available in the text

## 1 Introduction

Multiple sclerosis (MS) is a chronic demyelinating disease of the central nervous system (CNS), whereas presumed pathogenesis includes autoimmunity and heredity, characterized by demyelination, localized areas of inflammatory infiltration, axonal loss, and gliosis in the brain and spinal cord.^[1]^ It typically begins between the ages of 20 and 40 years, whereas women are affected approximately twice as often as men.^[2]^ People with MS have significant physical and cognitive impairments with some common symptoms: ataxia, bladder discomfort, visual impairment, and so on.^[3]^ There are 4 traditional recognized phenotypes: relapsing-remitting MS (RRMS), primary progressive MS (PPMS), secondary progressive MS (SPMS), and progressive relapsing MS (PRMS); clinically isolated syndrome (CIS) and radiologically isolated syndrome (RIS) were added to a revised standard in 2013 based on the progress of biomarkers, magnetic resonance imaging (MRI) imagines, and clinical symptoms.^[4]^ Nearly 85% of patients of MS belong to RRMS, the majority of whom do eventually progress into SPMS in a median time about 19 years if untreated.^[5]^ The McDonald series have been widely used in the diagnosis of MS in the past few years, which had been updated in 2017 for the need of an earlier and more accurately diagnose based on more pathological details.^[6–8]^

The global Atlas of MS had been updated in 2013 as a joint project of the Multiple Sclerosis International Federation (MSIF) and the World Health Organization (WHO). It suggested that patients with MS rose from 2.1 million to 2.3 million between 2008 and 2013, whereas the incidence in North America (more than 100 people per 100,000) and Australia (more than 60 people per 100,000) were obviously higher than other WHO regions.^[9,10]^ Latest local investigation and evaluation in Malaysia, Japan, Latin America, and so on also showed increasing prevalence in the past few years.^[11–14]^ Furthermore, global and regional economic impact assessment of MS reported a significant rise in medical investment, social support, and intangible costs in recent years. It is predicted that the total annual health sector costs for MS will reach $2.0 billion by 2031 just in Canada.^[15–18]^ Although the various quality of original research, prolonged survival, and developing diagnostic criteria may have an impact on the results of the evaluation, the increase in prevalence is a warning.

The past decade has shown a revolution in treatment options of MS, but there is no curable treatment available for MS. Current therapeutic strategy is aimed at reducing the risk of relapses and potentially disability progression.^[19]^ Disease-modifying therapies (DMTs) series are the most common treatments consisting of kinds of drugs such as Interferon beta, Glatiramer acetate, Fingolimod, and so on, but lack of convincing evidence of slowing down or preventing disease progression in SPMS patients.^[20]^ Some latest guidelines (2018 ECTRIMS/EAN, 2014 NICE) focusing on disease-modifying treatment and other options have provided detailed recommendations for MS, whereas evidence quality and therapy efficacy still create many uncertainties and weak recommendations in the treatment of MS.^[21,22]^

Siponimod (BAF312) is a second-generation sphingosine-1-phosphate (S1P) receptor modulator that is similar to Fingolimod, but with a significantly shorter elimination, which prompts an alternative option with higher security.^[23]^ Findings from preclinical studies suggest that it might prevent synaptic neurodegeneration and promote remyelination in the CNS.^[24,25]^ Several clinical randomized placebo-controlled trials in the past decade had confirmed its promising prospects for the treatment of MS, especially for SPMS. But various doses, disease subtypes, and outcomes in the original studies had brought difficulties to understand the objective effect of siponimod for MS, whereas some adverse events (increased alanine aminotransferase, macular edema, hypertension, and so on) were reported more frequently than the placebo group, which prompted for potential safety risks.^[26–28]^ Apart from this, the limited sample size reduces the test efficiency, which may lead to wrong results.

However, there is no systematic assessment of published data of siponimod for MS. Thus, we intend to conduct this systematic review and meta-analysis hoping to evaluate the efficacy and safety of siponimod for MS.

## 2 Method

### 2.1 Protocol and registration

This systemic review had applied for registration in the PROSPERO international prospective register of systematic review (PROSPERO#CRD42018112721) when we started to search related studies. We prepare to conduct it according to the guidelines set forth in the Cochrane Handbook for the Systematic Reviews of Interventions and Preferred Reporting Items for Systematic Review and Meta-Analysis (PRISMA). No requirement of ethical approval and informed consent is needed because it is a secondary study.

### 2.2 Search strategies

We will conduct a systematic search without language and year restrictions to identify all relevant published and unpublished Clinical controlled trials. The following electronic databases will be searched: PubMed, EMBASE, Cochrane Library, Clinical Trials, and Cochrane Central Register of Controlled Trials (CENTRAL). The keywords of search strategies are “siponimod,” “BAF312,” and “BAF-312.” In addition, we will manually retrieve congress reports and conference proceedings. References of included study will also be traced back to find potential studies. To identify other relevant study data, we will contact the authors of published studies for incomplete data or contact authors of unpublished manuscripts to ask whether they are willing to provide their unpublished data. Detailed search strategies could be found in Appendix 1.

### 2.3 Eligibility criteria

We will include studies which meet the following criteria: the study disease was MS (diagnosed by McDonald series^[6–8]^) with no restrictions on the comorbidities of patients; the study intervention was only siponimod with no restrictions on the dose or delivery route; the study control was placebo with no restrictions on placebo type; the study design was clinical randomized controlled trials.

Studies will be excluded if 1 of the following conditions is met: the study disease was not MS; the intervention of original research was not siponimod or siponimod associated with other drugs; the study control was not a placebo; the study design did not match such as animal experiment, review, comment; and the research data were missing too much or were not available.

### 2.4 Study selection and data extraction

Titles and abstracts of the citations retrieved by the literature search will be screened independently for inclusion/exclusion by 2 review authors. We will select the full text of potentially relevant studies for further assessment. Any disagreement will be resolved by a third reviewer. Two other review authors will independently extract the related data using a data extraction form including research basic information, result data, and quality evaluation information. Any dispute will be discussed and resolved by a third reviewer. We will contact the author if the data is not fully reported.

### 2.5 Outcomes

Outcomes were initially identified by reading relevant literature combined with our clinical experience. The primary outcomes are annualized relapse rate (ARR), the percentage of patients with a prespecified number of confirmed disease progression (CDP), and adverse events. Relapse is defined as new, or worsening, pre-existing neurological symptoms with a change in Functional Score on Expanded Disability Status Scale (EDSS) score shown by the examining physician, and CDP is defined as an increase of at least 1.5 points on the EDSS scale for patients with a baseline score of 0, of at least 1.0 point for patients with a baseline score of 1.0 or more, and of at least 0.5 point for patients with a baseline score of 5.5 or more.^[29]^ The secondary outcomes include regularly measured MRI indicators: number of T1 gadolinium-enhancing lesions, number of new or newly enlarged T2 lesions, and brain volume change from baseline. We will record all the endpoints that reported in the original studies for a comprehensive analysis, although we do not mention some of them in this protocol.

### 2.6 Assessment of study bias

Two review authors will independently assess the potential bias of included randomized controlled trials using the Cochrane risk of bias tool. The tool for evaluating the risk of bias consists of 7 specific domains: sequence generation; allocation concealment; blinding of participants and personnel; blinding of outcome assessment; incomplete outcome data; selective outcome reporting; and other bias. Each study will be reviewed from these domains to assess their bias level. If there is any question, a third reviewer will solve it.

### 2.7 Strategy for data synthesis

Cochrane Systematic review software Review Manager (RevMan; Version 5.3) will be used to conduct meta-analysis. Dichotomous variables will be presented as relative risk (RR) and hazard ratio (HR) with 95% confidence intervals (CIs). Continuous variables will be presented as mean difference (MD) with 95% CIs. The heterogeneity between the included studies will be analyzed by Cochrane chi-square test (test level is α = 0.05), and *I*^2^ will be used to quantitatively determine the heterogeneity. Substantial heterogeneity is defined as *P* < .05 in and *I*^2^ > 50%. When substantial heterogeneity exists, a random-effects model will be used; otherwise, the fixed-effect model will be used. If data are too heterogeneous to pooling of effect sizes in a meaningful or valid way, we will use a narrative approach to synthesize the data.

### 2.8 Preset subgroup analysis

Subgroup analysis will be conducted to find more potential information based on preset criteria as shown below: different sex of patients; different subtypes of MS; different doses of siponimod; and different duration of treatment. The subgroup analysis may be adjusted according to the results of pooling.

### 2.9 Publishing bias and sensitivity analysis

If there are more than 10 studies for data synthesis, we will create and examine a funnel plot to explore possible publication bias. Sensitivity analysis will be carried out by excluding trials of low/moderate methodological quality or researches with significant large/small effect values, and results will be presented and compared with overall findings.

## 3 Discussion

This is the first systematic review and meta-analysis of the efficacy and safety of siponimod in the treatment of MS. Our evaluation results may form new clinical recommendations and provide a reference for further research.

There appears to be some difficulties and limitations according to our previous work, such as limited number of randomized controlled trials, dose inconsistency for different studies, complex disease subtype, changing outcomes of different research in the past decades, which may have an obstacle to our data pooling. But it probably prompts some more reliable conclusions and direction of future clinical studies to some extent. We hope to provide a timely and reliable evaluation of siponimod which may be a new survival guarantee for MS patients.

## Author contributions

Y.M.S contributed to study concept and design, Y.F.L wrote the first draft and other authors had gave some suggestions for modification.

## Supplementary Material

Supplemental Digital Content
