# Supplementary material for: Efficacy and safety of siponimod for multiple sclerosis: Protocol for a systematic review and meta-analysis
Source: Medicine (Baltimore). 2019 Aug 23;98(34):e15415. doi: 10.1097/MD.0000000000015415 (PMC6716697; doi:10.1097/MD.0000000000015415)
Supplement: Supplemental Digital Content [file medi-98-e15415-s001.docx]

**Appendix 1**

**Search strategy in different database.**

**PubMed:**

**#1((siponimod[Title/Abstract])**

**#2 BAF312[Title/Abstract])**

**#3 BAF-312[Title/Abstract]**

**#4 #1 OR #2 OR #3**

**EMBASE:**

**#1 'siponimod'/exp**

**#2 siponimod:ti,ab,kw**

**#3 baf312:ti,ab,kw**

**#4 'baf-312':ti,ab,kw**

**#5 #1 OR #2 OR #3 OR #4**

**Cochrane Library:**

**#1 (siponimod):ti,ab,kw**

**#2 (BAF312):ti,ab,kw**

**#3 (BAF-312):ti,ab,kw**

**#4 #1 OR #2 OR #3**
